# Self-supervised learning for human activity recognition using 700,000 person-days of wearable data

**DOI:** 10.1038/s41746-024-01062-3

**Published:** 2024-04-12

**Authors:** Hang Yuan, Shing Chan, Andrew P. Creagh, Catherine Tong, Aidan Acquah, David A. Clifton, Aiden Doherty

**Affiliations:** 1 Nuffield Department of Population Health, University of Oxford, Oxford, UK; 2 Big Data Institute, Li Ka Shing Centre for Health Information and Discovery, University of Oxford, Oxford, UK; 3 Department of Computer Science, University of Oxford, Oxford, UK; 4 Department of Engineering Science, University of Oxford, Oxford, UK

**Keywords:** Computer science, Diagnostic markers, Epidemiology

## Abstract

Accurate physical activity monitoring is essential to understand the impact of physical activity on one’s physical health and overall well-being. However, advances in human activity recognition algorithms have been constrained by the limited availability of large labelled datasets. This study aims to leverage recent advances in self-supervised learning to exploit the large-scale UK Biobank accelerometer dataset—a 700,000 person-days unlabelled dataset—in order to build models with vastly improved generalisability and accuracy. Our resulting models consistently outperform strong baselines across eight benchmark datasets, with an F1 relative improvement of 2.5–130.9% (median 24.4%). More importantly, in contrast to previous reports, our results generalise across external datasets, cohorts, living environments, and sensor devices. Our open-sourced pre-trained models will be valuable in domains with limited labelled data or where good sampling coverage (across devices, populations, and activities) is hard to achieve.

## Introduction

Cost-effective wearable sensors have gained increasing interest for their potential to revolutionise healthcare owing to their wide range of applications, including fitness and wellness tracking, remote patient monitoring^1,2^, early disease detection^3,4^, real-time clinical trials^5–7^, large-scale population health studies^8–11^, and personalised medicine^12^. Consumer-grade devices allow users to obtain summary movement and behaviour metrics such as sleep quality, sedentary time, pace, and step counts. Critical to their effectiveness is the use of reliable algorithms to infer human activities from motion sensor data. However, methodological progress in human activity recognition has been constrained by the limited availability of large representative *labelled* datasets.

Contrary to fields that have benefited from an explosion of data and subsequent methodological leaps, such as computer vision^13–18^ and natural language processing^19–22^, wearables-based human activity recognition research still relies on very small datasets, the majority of which are collected in an artificial setting (e.g., participants following a predefined script in a lab environment and under supervision). Further, this small-data limitation confounds research findings involving data-hungry deep learning methods; for example, there exist empirical studies^23,24^, suggesting that deep learning methods such as DeepConvLSTM^25^ did not significantly improve upon more conventional methods relying on simple statistics of the sensor signal.

In this paper, we leverage the UK Biobank accelerometer dataset to realise the full potential of deep learning methods for activity recognition. The UK Biobank is a unique large-scale study that recruited roughly half a million participants, of which more than 100,000 wore a wrist accelerometer for 7 days in their usual environments (as opposed to lab settings), amounting to over 700,000 person-days (and many terabytes) of free-living, 24/7 human motion data.

In order to make use of this *unlabelled* dataset, we build upon recent advances in self-supervised learning, which have shown great results in this regard, with popular examples such as GPT^26^. A suite of self-supervised learning methods have been explored for wearable sensor data with success, including multi-task self-supervision^27^, masked reconstruction^28^, contrastive learning^18,29,30^, and bootstraping^31,32^. A recent benchmark provided a comprehensive assessment of existing self-supervised approaches for human activity recognition and concluded that multi-task self-supervision could learn the most generic features applicable to different downstream tasks^33^. Existing methods either used the same data for pre-training and fine-tuning or were only trained on datasets with a small size (*n* = 100), a limiting factor for the generalisability of the pre-trained models. By applying multi-task self-supervision on a large unlabelled dataset with three simple tasks, *arrow of time*, *permutation*, and *time warping*^27,34^, we showed for the first time that a pre-trained model that could generalise to a wide range of downstream activity recognition datasets important for clinical and health applications.

Our main contributions are:

- We demonstrate the application of multi-task self-supervised learning on a tera-scale wearables dataset to realise the full potential of deep learning in building state-of-the-art activity recognition models. We discuss engineering challenges in training on large and high-dimensional sensor data and other technical considerations.
- In contrast to previous works, we conduct a more realistic evaluation of the utility of self-supervised human activity recognition by factoring in common issues seen in practical use cases of pre-trained models such as domain shift and task shift^35^. In particular, our models show consistent outperformance on *external* datasets.
- We release our pre-trained models to enable the digital health research community to build high-performing models for their own use cases. Our models will be especially useful in domains with limited data.

## Results

**Fig. 1:**
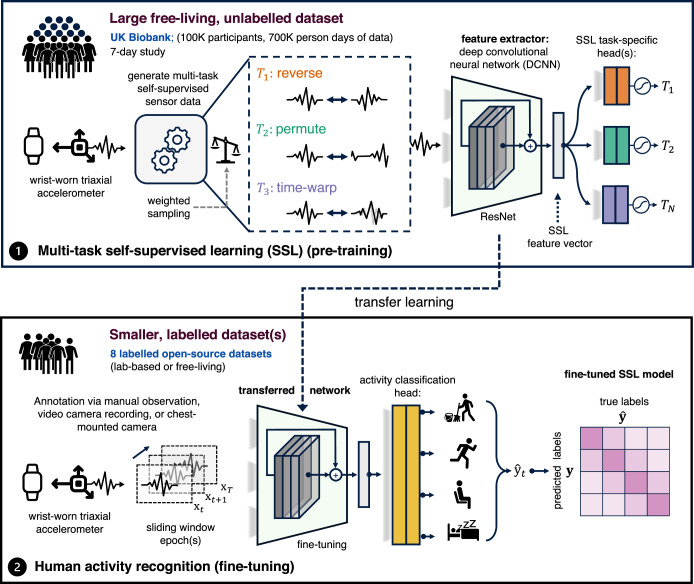
Overview of the proposed self-supervised learning pipeline. Step 1 involves multi-task self-supervised learning on 700,000 person-days of data from the *UK Biobank*. In step 2, we evaluate the utility of the pre-trained network in eight benchmark human activity recognition baselines via transfer learning. Reproduced and modified with permissions from ref. ^37^.

Figure 1 provides a schematic overview of our paper: first, we applied multi-task self-supervised learning to pre-train a deep convolutional neural network on 700,000 person-days of free-living accelerometer data from the UK Biobank; second, the pre-trained network is evaluated via transfer learning on eight benchmark datasets to assess representation quality on various activity types and populations.

### Weighted single-task training

**Fig. 2:**
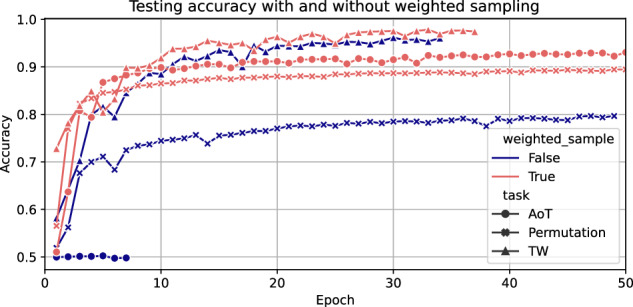
Accuracy test curves for training four self-supervised tasks individually using 1000 subjects from the UK Biobank with and without weighted sampling. The patience for early-stopping was five epochs.

When training individual pretext tasks, we found that without weighted sampling, all the tasks had worse convergence behaviour (Fig. 2). The performance degradation was most pronounced for the AoT and permutation. The test performance for the AoT stayed at the random chance level, and the test performance for permutation dropped ~10% points without weighted sampling.

### Multi-task self-supervised learning

To investigate how different self-supervision configurations perform in three downstream datasets, we picked one large (*Capture-24*), medium (*Rowlands*), and small (*Opportunity*) dataset for evaluation. We trained different tasks both individually and jointly using 1000 subjects from the *UK Biobank*, then we fine-tuned the models on the subsequent human activity recognition benchmarks (Table 1).

**Table 1:** Downstream human activity recognition performance (subject-wise F1 ( ± SD)) for different self-supervised task combinations using 1000 UK Biobank participants

AoT	Permutation	TW	Capture-24	Rowlands	Opportunity
			*n* = 573k	*n* = 36k	*n* = 3.9k
Single task					
*✓*	✗	✗	0.671 ± 0.094	0.565 ± 0.120	0.582 ± 0.054
✗	*✓*	✗	0.721 ± 0.093	0.783 ± 0.099	0.588 ± 0.076
✗	✗	*✓*	0.715 ± 0.093	0.776 ± 0.110	0.584 ± 0.064
Multi-task					
✗	*✓*	*✓*	0.714 ± 0.094	0.755 ± 0.103	0.587 ± 0.070
*✓*	✗	*✓*	0.719 ± 0.094	0.762 ± 0.102	0.530 ± 0.071
*✓*	*✓*	✗	0.718 ± 0.092	0.781 ± 0.101	0.502 ± 0.081
*✓*	*✓*	*✓*	0.718 ± 0.095	0.770 ± 0.102	0.482 ± 0.078

*N* is the number of samples.

The differences between different self-supervision combinations on large datasets (*Capture-24* and *Rowlands*) was smaller than that of the smaller dataset (*Opportunity*). There was no clear best-performing configuration, and thus, for ease of comparison, we chose to use all tasks in pre-training for the remaining experiments. In addition, training more tasks together might yield the most general representation for different downstream datasets.

### Downstream performance—human activity recognition

Table 2 summarises the F1 and Kappa scores for eight human activity recognition datasets. The random forest models outperformed the deep learning models trained from scratch for all except the *Capture-24* dataset, which is the largest labelled dataset in our evaluations (Table 4). The performance gap between random forest and training from scratch was the largest in smaller datasets. Meanwhile, pre-trained models outperformed the models trained from scratch and random forest in all eight datasets. Fine-tuning all layers was better than fine-tuning just the fully connected layers after the ConV layers.

**Table 2:** Subject-wise F1 and Kappa (*κ*) for downstream human activity recognition tasks (mean ± SD) using 100,000 participants for pre-training

			ResNet			
Data		Random forest	Trained from scratch	Fine-tune self-supervised		Improvement
				After ConV layers	All layers	
Capture-24	F1	0.694 ± 0.099	0.708 ± 0.094	0.723 ± 0.097	0.726 ± 0.093	2.5%
	*κ*	0.683 ± 0.101	0.703 ± 0.092	0.718 ± 0.090	0.737 ± 0.087	4.8%
Rowlands	F1	0.700 ± 0.090	0.696 ± 0.106	0.724 ± 0.081	0.796 ± 0.093	14.4%
	*κ*	0.830 ± 0.086	0.810 ± 0.098	0.850 ± 0.062	0.874 ± . 073	7.9%
WISDM	F1	0.711 ± 0.149	0.684 ± 0.123	0.759 ± 0.121	0.810 ± 0.127	18.4%
	*κ*	0.715 ± 0.153	0.685 ± 0.124	0.758 ± 0.121	0.809 ± 0.126	18.1%
MJFF-LR	F1	0.590 ± 0.136	0.327 ± 0.103	0.677 ± 0.094	0.755 ± 0.109	130.9%
	*κ*	0.653 ± 0.126	0.347 ± 0.128	0.715 ± 0.091	0.817 ± 0.080	135.4%
REALWORLD	F1	0.731 ± 0.119	0.705 ± 0.062	0.764 ± 0.052	0.792 ± 0.075	12.3%
	*κ*	0.680 ± 0.142	0.638 ± 0.079	0.703 ± 0.063	0.739 ± 0.086	15.8%
Opportunity	F1	0.416 ± 0.185	0.383 ± 0.124	0.570 ± 0.078	0.595 ± 0.085	55.4%
	*κ*	0.318 ± 0.206	0.238 ± 0.154	0.435 ± 0.092	0.471 ± 0.104	97.9%
PAMAP2	F1	0.753 ± 0.093	0.605 ± 0.086	0.725 ± 0.054	0.789 ± 0.054	30.4%
	*κ*	0.744 ± 0.101	0.596 ± 0.086	0.717 ± 0.057	0.769 ± 0.059	29.0%
ADL	F1	0.764 ± 0.180	0.414 ± 0.179	0.645 ± 0.107	0.829 ± 0.101	100.0%
	*κ*	0.720 ± 0.199	0.368 ± 0.198	0.654 ± 0.123	0.849 ± 0.113	130.7%

The relative improvement compares the performance between the model that is trained from scratch and fine-tuning using all the layers. Datasets are ranked by subject number from large to small.

The most significant improvement using pre-training was seen on the small datasets. Conversely, the benefit of self-supervised learning was more modest for larger datasets. In *Capture-24*, the F1 improvement was 2.5% when comparing the model with and without self-supervised pre-training. Nonetheless, with self-supervised pre-training, the median relative F1 improvement was 18.4% when compared to the same network trained from scratch and 8.5% when compared to the random forest model.

### Transfer learning using labelled pre-training

Even though supervised pre-training can boost the learning outcome substantially more than training from scratch (Table 2 vs Table 3), self-supervised pre-training without labels could outperform supervised pre-training when using *Rowlands* and *Capture-24* as the source data.

**Table 3:** Transfer learning (subject-wise F1 ( ± SD)) performance comparison between supervised pre-training with self-supervised pre-training

	Source data				
Target data	Rowlands		Capture-24		UK Biobank
	Supervised	Self-supervised	Supervised	Self-supervised	Self-supervised
Capture-24	0.707 ± 0.094	0.709 ± 0.094	–	0.707 ± 0.094	0.726 ± 0.093
Rowlands	–	0.734 ± 0.082	0.728 ± 0.094	0.730 ± 0.084	0.796 ± 0.093
WISDM	0.680 ± 0.109	0.702 ± 0.123	0.715 ± 0.119	0.723 ± 0.121	0.810 ± 0.127
MJFF-LR	0.331 ± 0.159	0.468 ± 0.161	0.616 ± 0.127	0.601 ± 0.114	0.755 ± 0.109
REALWORLD	0.712 ± 0.086	0.737 ± 0.105	0.759 ± 0.070	0.771 ± 0.061	0.792 ± 0.075
Opportunity	0.536 ± 0.019	0.539 ± 0.018	0.547 ± 0.043	0.547 ± 0.042	0.595 ± 0.085
PAMAP2	0.677 ± 0.082	0.689 ± 0.078	0.678 ± 0.118	0.725 ± 0.725	0.789 ± 0.054
ADL	0.634 ± 0.182	0.701 ± 0.111	0.768 ± 0.169	0.754 ± 0.159	0.829 ± 0.101

### Ablation studies

#### Varying labelled data in the downstream

**Fig. 3:**
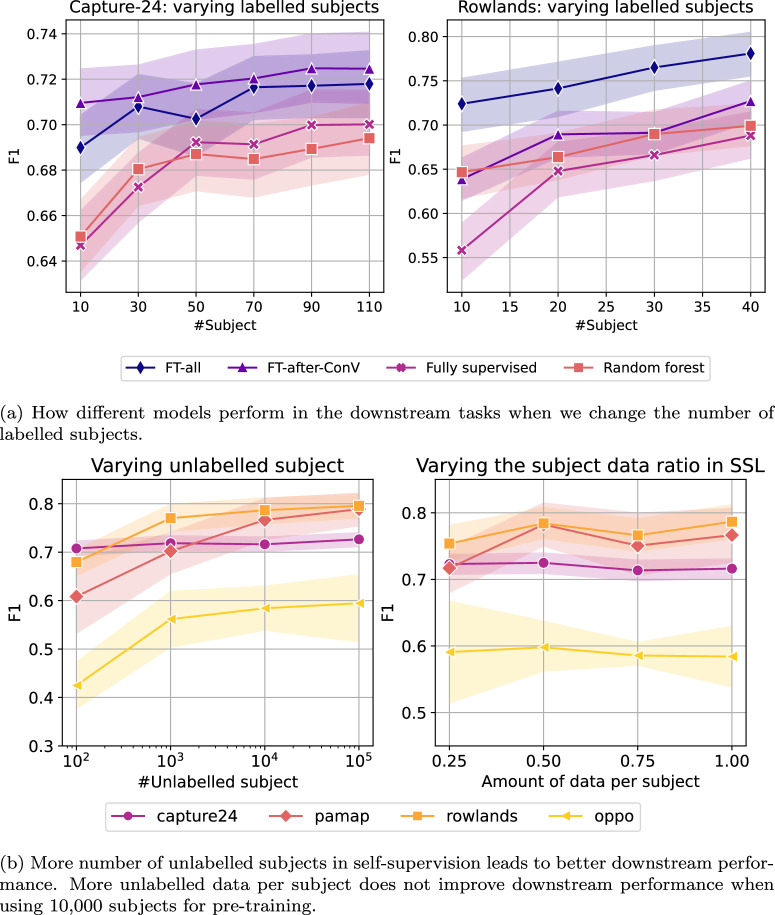
The association between data volume and model performance. The impact of varying amount of labelled data in fine-tuning (**a**) and unlabelled data in self-supervision (**b**) on subsequent human activity recognition performance. Mean F1 ± SD are plotted.

We observed that pre-trained models did well regardless of the number of labelled subjects in two downstream datasets (Fig. 3a). However, fully supervised and random forest models were more susceptible to the number of labelled subjects. The performance gain for having more labelled subjects was roughly linear with respect to the number of subjects included with a greater increase when we had fewer labelled subjects.

#### Varying unlabelled data in the pre-training

We also found that the downstream human activity recognition performance appeared to increase linearly with respect to the number of unlabelled subjects on a log scale (Fig. 3b, left). The self-supervision performance boost with more unlabelled subjects pre-training was most significant in the smallest dataset, *Opportunity*. Furthermore, if the number of participants is fixed at 10,000 in pre-training, the data ratio included per subject did not significantly influence the downstream performance. Notably, the downstream performance did not degrade more than 10% in F1 even when we reduced the the amount of data per subject from 100 to 25%. (Fig. 3b, right).

### Understanding the representation

#### Cluster analysis

**Fig. 4:**
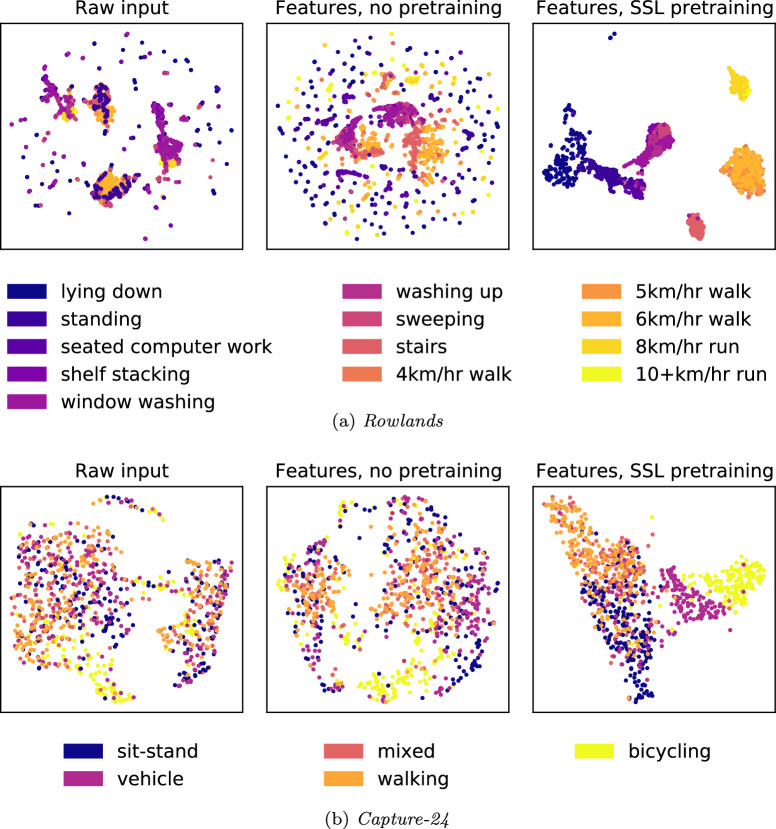
Cluster analysis on raw inputs, untrained features and self-supervised-pre-trained features. Colour gradients were used to denote activity intensities (**a**: *Rowlands*, **b**: *Capture-24*).

We used UMAP^36^ with default parameters for low-dimensional projections for visualisation. This was applied to the raw inputs, untrained features, and self-supervision-derived features *without fine-tuning*. Results for two of the downstream datasets are shown in Fig. 4, and the remaining results can be found in Supplementary Fig. 2. Across all datasets, we observed that the self-supervision-derived features were better at clustering similar activities (e.g., walking, stair climbing vs sitting, writing, typing) as well as their intensities (e.g., lying down, sitting, standing vs jogging, sports), exhibiting better intra-class compactness and inter-class separability.

#### Feature interpretation

Next, we visualised two exemplary pretext self-supervised task predictions in the presence of repetitive low- and high-intensity activities: shaking hands (Supplementary Fig. 4a) and playing tennis (Supplementary Fig. 4b). During tennis playing, a repetitive high, intensity activity, relevance scores tended to highlight the moments around the natural movements of swinging and hitting the tennis ball (Supplementary Figs. 4b and 5). When performing a repetitive low-intensity activity experiment, for example, shaking hands (Supplementary Fig. 4a), layer-wise relevance propagation appeared to also identify the intensity and natural signal periodicity as indicative of the original activity. In contrast, for augmented signals, our model attributed more during periods of visually unrealistic motion dynamics, such as unnatural fragmentation in activity frequency or synchronisation mismatches between sensor axes. Interestingly, stationary movement periods were not relevant for detecting the pretext tasks.

Finally, we empirically compared the faithfulness of the explainable AI algorithms investigated and the combination of various layer-wise relevance propagation parameters, using sample-masking experiments for a random subset of 1000 (out-of-sample) subjects in the *UK Biobank*. Most explainable AI models consistently demonstrated the ability to identify relevant patterns for discriminating transformed samples from the original raw data when compared against a random model.

## Discussion

Our work has shown that self-supervised pre-training consistently improved downstream human activity recognition, especially in small datasets, reducing the need for labelled data. The self-supervised representations generalise well across a range of external datasets, tasks, devices, health statuses, and populations, a key aspect in activity monitoring for clinical use. Our work represents the most robust human activity recognition foundational model to date, as it is trained on a much larger and more diverse dataset than previous efforts in this space. The scale of *UK Biobank* is several orders of magnitude greater than the largest datasets used by the current state-of-the-art such as the *Fenland* data^9^ (*n* = 160,000). In addition, not just big in size, but *UK Biobank* is much more diverse as it contains hundreds, if not thousands, of natural human activities—a crucial aspect regarding the training data and feature generalisation. Indeed, the obtained pre-trained model has already been used with success in enhancing digital monitoring for a clinical population with motor impairment^37^ and epidemiological research^38,39^.

With our pre-trained models, one can obtain a highly competitive activity recognition model with a small amount of labelled data, a feature important for clinical studies where labelled data is expensive to acquire. In contrast, previous studies applied self-supervised training and fine-tuning only on the same data sources^24,27,40^, making it necessary to pre-train on every new dataset in practical applications. A recent attempt has been made to systematically evaluate the effect of self-supervised techniques by pre-training on Capture-24, providing a good baseline for the performance evaluation in human activity recognition^33^. However, pre-training on Capture-24 with roughly 100 participants will not be able to characterise the impact of self-supervised pre-trained models. The representation quality is superior if the pre-training is done on datasets with richer population characteristics. Our pre-trained network can serve as a foundational human activity recognition model that removes the need to pre-train on unseen datasets.

We found that the representation quality from self-supervision was always better than that of supervised learning in an apple-to-apple comparison when using *Rowlands* as the source of pre-training. Self-supervised learning with other modalities has also found that self-supervised pre-training can outperform supervised pre-training^18,41^. Pre-training on the *UK Biobank* yields the most significant improvement among all the self-supervised representations, as the human activity recognition performance is uniformly better than other pre-trained baselines (Table 3). The performance boost could be attributed to the large data volume, diverse activity classes and rich population characteristics of the *UK Biobank* over alternative pre-training datasets. Recent investigations on large language models have profiled the trade-off between model size and data volume when the compute budget is fixed^42^, suggesting model size is another important dimension for pre-training worthy of further investigation for human sensing data.

This study highlights new questions to prioritise in future. Due to a current lack of raw accelerometer datasets in different regions of the world, a limitation of our work is that the pre-training data (*UK Biobank*) consists mostly of Caucasians from the UK. A multi-modal representation that also includes electrocardiogram and other time-series wearable sensor data sources will also be important to consider when such datasets are collected in the future. Lastly, future work could also investigate the representation quality using more recent self-supervised learning approaches on free-living UK Biobank accelerometers^28,32,33,40,43^, in addition to multi-task learning. We attempted to use Autoencoder and contrastive learning for pre-training. However, we could not obtain high-quality representation using the UK Biobank^43^. We suspect this was mainly due to the difference between free-living and lab-based activity data, which can be further analysed to compare the performance of different self-supervised methods.

We have developed and evaluated a self-supervised deep neural network on large-scale activity tracking data. The features obtained improved on prior state-of-the-art performance across eight benchmark human activity recognition datasets. Our open-sourced model represents a foundation model that others can build upon for state-of-the-art human activity recognition applications. The improved physical activity measurement will help to understand better the influence of physical activity on different disease outcomes, especially for populations that have been under-represented in previous studies.

## Methods

We used tri-axial accelerometer data from wrist-worn activity trackers, which record acceleration on three orthogonal axes at a high sampling rate (e.g., 100 Hz). The main benefit of wrist-worn activity trackers is their high user compliance, resulting in days, if not weeks, of continuous recordings. Following ref. ^44^, we split the signals into windows of equal duration, effectively treating them as independent inputs to the human activity recognition models. We can then label each window with an activity class. Throughout this study, we linearly resampled all data to 30 Hz resolution and used ten-second-long windows to compare the downstream benchmarks fairly. The 30 Hz sampling rate was used because most human activities have a frequency less than 10 Hz. We used a sampling rate that is higher than the presumed Nyquist rate (20 Hz) to ensure that we did not lose any useful signal.

### Datasets

Our multi-task self-supervised training relied on the *unlabelledUK Biobank* dataset, which contains roughly 700,000 person-days of free-living activity data (>100,000 participants, 7 days of wear). The free-living aspect is important because the data can contain all sorts of activities, as opposed to lab data which are constrained to scripted activities only. The UK Biobank data (project ref 21/NW/0157) is covered by ethical approval from the NHS National Research Ethics.

For the subsequent activity recognition benchmarks, we considered eight external *labelled* datasets that vary in size (600–600,000 samples), activity classes (4–18 classes), devices (5 different brands), device placements (4 configurations), populations (differ in age, sex, and health status), and collection protocol (free-living, scripted, and lab settings). See Table 4 and Supplementary Table 1 for detailed dataset characteristics. Three datasets had license information, and five datasets had explained informed consent information (Supplementary Table 2). We removed the classes that were not present in all the subjects in small datasets with less than ten individuals during data cleaning. The Michael J. Fox Foundation Levodopa Response (MJFF-LR) study was included to assess the generalisability of our model in a clinical population with motor impairment, Parkinson’s disease in our case. For the MJFF-LR study, we only included data collected in a lab because not all the participants had free-living data. Finger-to-nose and repeated-arm movement tasks were also removed from MJFF-LR as these two activities were performed using both arms in alternation, but we only used the data from one arm. We further merged three walking classes into one.

**Table 4:** Wrist-worn accelerometer datasets used to evaluate the utility of self-supervised learning for human activity recognition tasks

Dataset	#Subjects	#Samples	#Classes	Environment	References
UK Biobank	~100K	6 B	Unlabelled	Free-living	^55^
Capture-24	152	573K	4	Free-living	^8^
Rowlands	55	36K	13	Lab	^56^
WISDM	46	28K	18	Semi free-living	^57^
MJFF-LR	28	12K	12	Lab	^58^
REALWORLD	14	12K	8	Lab	^59^
Opportunity	4	3.9K	4	Semi free-living	^60^
PAMAP2	8	2.9K	8	Lab	^61^
ADL	7	0.6K	5	Lab	^62^

Even though we reused existing datasets, we made our best effort to enumerate the license and consent information for all the included datasets, as our data involved human subjects. We observed that many open benchmark datasets that we used did not have suitable licensing or consent information, possibly due to the lack of data governance awareness at the time of the study.

### Multi-task self-supervised-learning

We considered three self-supervised tasks from ref. ^27^, which were first used in ref. ^34^ as data augmentation techniques. Eight transformations were included in the previous exploration of multi-task learning^27^. We chose arrow of time, permutation and time warping to maximise learning features related to human motion dynamics. Supplementary Methods Section explains why other transformations were not chosen.

Arrow of time (AoT) flips the signal along the time axis, effectively playing the signal in reverse. Permutation breaks the signal into chunks and shuffles them. We set the number of chunks to four and the minimum length of each chunk to at least ten timestamps. Time warping (TW) stretches and compresses arbitrary segments of the signal, effectively slowing down and speeding up the signal randomly.

Following ref. ^27^, we treated each of the tasks as a binary problem predicting whether a transformation has been applied. In the multi-task learning (MTL) setting, not all the tasks might benefit human activity recognition when trained jointly, so we assessed how different task combinations could influence the downstream performance. We computed the cross-entropy loss for each task and weighed all the tasks equally in the loss calculation.

#### Weighted sampling

Motion data collected in the real world contains large portions of low movement periods that are less informative (Supplementary Fig. 1), which is an issue for our self-supervised tasks as static signals remain virtually unchanged after the transformations. We found it crucial to perform weighted sampling for improved training stability and convergence: during training, we sample the data windows in proportion to their standard deviation so as to give more weight to high-movement periods.

### Network training

We adapted a ResNet-V2 with 18 layers and 1D convolutions^45^ for the main trunk (feature extractor), totalling 10M parameters. The learned feature vector was of size 1024. All the tasks shared the same feature extractor. Then, we attached a softmax layer for each of the self-supervised tasks. In the downstream evaluation, we added a fully connected (FC) layer of size 512 in between the feature extractor and softmax readout. The network structure was fixed for all the downstream evaluations.

For self-supervised learning, we load up to four subjects from the *UK Biobank* at each iteration. For each subject, we first sampled one day out of the week-long data, from which we again sampled 1500 10-s windows to make up a training batch. Self-supervised transformations were then applied to the batch of data. Since the axis orientation differs between device manufacturers, we used random axis swaps and rotations to augment the training data to embed this invariance into our models. For optimisation, we used Adam^46^ with a learning rate of 1e-3. To account for large batch sizes, 1500 × 4 = 6000, we applied linear scaling for the learning rate with five epochs as burn-in^47^. We distributed the network training over four Tesla V100-SXM2 with 32 GB of memory. It took about 420 GPU hours to train the MTL model (about 20 epochs). We used an 8:2 ratio for the train/test split for all the self-supervised experiments. For fine-tuning, we used the same training setup as the pre-training where possible, except for the batch size, which was re-adjusted depending on the size of each dataset.

### Evaluation—human activity recognition

To evaluate the downstream human activity recognition performance, we used held-one-subject-out cross-validation for the datasets that had <10 subjects. We additionally removed activity classes not done by all the subjects in these small datasets. For datasets with ≥10 subjects, we used five-fold subject-wise cross-validation instead. Each cross-validation had a 7:1:2 split ratio for train/validation/test sets. We used early-stopping with a patience of five to avoid over-fitting. For training runs that did not converge, we reported the best performance after using three different random seeds for weight initialisation.

After the network was trained on the *UK Biobank* using ~100,000 participants, we further fine-tuned the network on the eight labelled downstream datasets to perform human activity detection using two approaches: (1) fine-tuning all the layers (2) freezing the trunk (feature extractor) and fine-tuning only the FC layers in the end. We also report the model performance for a network of the same architecture but fully trained from scratch, and a strong random forest model with tried-and-tested time series features, which has often been neglected in baseline model comparisons^8,48–50^. See the Supplementary Methods Section for the list of features used.

In addition, a shared implementation was introduced for our network training, model evaluation and preprocessing. Differences in experiment setup such as training rates, regularisation and data augmentation can lead to inconsistent results^51^. A unified evaluation framework would ensure a fair comparison between different baseline models. Our evaluation framework contrasts with previous work, where there is no fixed evaluation protocol across the benchmark datasets, making it hard to compare model performance with the current state-of-the-art. The results produced in our paper would serve as the baseline for future human activity recognition research.

#### Transfer learning

Pre-training on a larger labelled dataset and fine-tuning on a smaller dataset is a common technique in practical application that has been under-reported as a baseline for self-supervision. The success of transfer learning, however, depends on how similar the source and target datasets are. Hence, we included experiments using the two largest labelled datasets, *Capture-24* and *Rowlands* for pre-training, which were then fine-tuned on other labelled datasets.

#### The benefits of data volume

In the ablation studies, we investigated how the downstream performance differs on two axes, the amount of labelled data and the amount of unlabelled data. Concretely, we gradually increase the number of labelled subjects in both *Capture-24* and *Rowlands* in the downstream evaluation to assess whether our pre-trained model can still do well in a limited-data regime. In terms of unlabelled data, we experimented with pre-training that had 100 to 100,0000 participants with one order of magnitude increment. We also varied the amount of unlabelled data per subject from 0.25 to 1 using 10,000 participants. A data ratio of 0.25 means that if one day of data per subject was used previously, then only 6 h of data per subject would now be used for training. Investigating how unlabelled data influences downstream performance will guide how much data one needs to have to obtain an effective self-supervised model for human activity recognition.

### Understanding network representation

#### Contextualising layer-wise relevance propagation

We applied layer-wise relevance propagation (LRP) to visually investigate the signal characteristics relevant for detecting the pretext tasks^52,53^. It is inherently more difficult to visually interpret attribution heatmaps generated through Explainable AI (XAI) frameworks on time-series signals. To overcome this lack of visual ground truth, we devised a set of simple contextual experiments to evaluate our LRP attribution results. Using the same accelerometer as the *UK Biobank*, we recorded a participant performing two activities under video observation: (1) low-intensity scripted (hand-shaking) and (2) high-intensity unscripted (playing tennis). We acquired a ground truth (the context) for the accelerometer activity through the time-synced video observations, enabling a better visual interpretation of the sensor-based characteristics attributed as relevant for detecting different pretexts. Holistic interpretations were formed based on visualising the raw sensor signal, its analogues time-frequency representation through continuous wavelet transform (CWT) scalograms^54^, as well as the time- and pretext task-localised LRP relevance scores, all with respect to observing the concurrent video recordings. Details on the XAI contextual LRP (cLRP) framework are described in the Supplementary Methods Section.

### Reporting summary

Further information on research design is available in the Nature Research Reporting Summary linked to this article.

### Supplementary information

Supplemental material

Reporting Summary

## Data Availability

The UK Biobank accelerometer dataset used for training can be requested by application (https://www.ukbiobank.ac.uk/enable-your-research/register). All the other evaluation datasets can be downloaded via https://github.com/OxWearables/ssl-wearables. The MJFF-LR study can be accessed via https://www.synapse.org/#!Synapse:syn20681023/wiki/594686 after registration. The Rowlands dataset can be requested by contacting Alex Rowlands directly.

## Code availability

The underlying code for for this study is available in OxWearables/ssl-wearables on Github and can be accessed via this link https://github.com/OxWearables/ssl-wearables.
