# Supplementary material for: Self-supervised learning for human activity recognition using 700,000 person-days of wearable data
Source: NPJ Digit Med. 2024 Apr 12;7:91. doi: 10.1038/s41746-024-01062-3 (PMC11015005; doi:10.1038/s41746-024-01062-3)
Supplement: Supplementary file 1 — Supplemental material [file 41746_2024_1062_MOESM1_ESM.pdf]

Supplementary Information

1. Methods

1.1. Datasets

Supplementary Table 1: Dataset characteristics

Dataset	Age<br>Mean (± SD)	Sex<br>Female (Male)	Health condition	Device	Placement	Country
UK Biobank	56.0 ± 7.8	58,275 (45,397)	Healthy	Axitivity (AX3)	Dominant	United Kingdom
Capture-24	18 - 91	99 (53)	Healthy	Axitivity (AX3)	Dominant	United Kingdom
Rowlands	40 - 63	37 (23)	Healthy	Genea	Both	United Kingdom
WISDM	-	-	Healthy	LG G Watch	Dominant	USA
MJFF-LR	62.5 ± 8.8	9 (19)	Parkinson's disease	GeneActiv	Wrist of most impairment	USA
REALWORLD	18 - 25	-	Healthy	LG G Watch R	Dominant	USA
Opportunity	-	-	Healthy	Custom inertial measurement units (IMUs)	Dominant	Switzerland
PAMAP2	27.2 ± 3.3	1 (8)	Healthy	Colibri inertial measurement units (IMUs)	Dominant	Germany
ADL	-	-	Healthy	Non-specified	Right	Italty

Supplementary Table 2: The license, consent and device used for each dataset

Dataset	Personal Info	Licence	Consent
UK Biobank	✗	Non-exclusive licence (but not ownership rights) for permitted purpose only	Informed consent for research
Capture-24	✗	CC Attribution	Informed consent for research
Rowlands	✗	Non-specified	Informed consent for research
WISDM	✗	Non-specified	Non-specified
MJFF-LR	✗	Non-specified	Informed consent for research
REALWORLD	✗	Non-specified	Non-specified
Opportunity	✗	Non-specified	Non-specified
PAMAP2	✗	Non-specified	Informed consent for research
ADL	✗	Free for research	Non-specified

1.2. Multi-task learning self-supervision

The original multi-task self-supervised learning for human activity recognition included a list of eight transformations: noised, scaled, rotated, negated, horizontally flipped, permuted, time-warped, and channel-shuffled [1]. In our multi-task setting, we used horizontally flipped (arrow of time), permuted, and time-warped for learning the features related to human motion dynamics. The success of multi-task learning requires a good synergy between the tasks included. To facilitate the design for future multi-task learning efforts, We explain below why the other transformations were not used:

- Noised: A noised signal tends to produce poor quality features leading to performance worse than random chance level [1];
- Scaled: Different devices likely have different absolute values, and thus scaling the value of the signals is less relevant for human motion dynamics;
- Rotated: We used rotation in data augmentation to produce a direction-invariant network;
- Negated: Negation is a form of rotation, making it redundant;
- Channel-shuffling: We used channel-shuffling in data augmentation to produce a direction-invariant network.

1.3. Feature engineering

The list of hand-crafted features that were extracted for random forest:

- Mean, standard deviation, and range for each axis.
- Correlation between each axis pair.
- Euclidean norm, its mean, standard deviation, range, median absolute deviation, kurtosis, and skew.
- The top two dominant frequencies in the power spectrum.

1.3.1. Explainable AI Framework for Time-Series Prediction

Holistic visual interpretation of the AoT predictions were determined by (1) visualising the raw data, (2) its time-frequency representation using the (discretised) continuous wavelet transform (CWT) and (3) assessing the time-localised LRP attribution.

The Continuous Wavelet Transform:. The CWT is a method used to measure the similarity between a signal and an analysing function (in this case the Morlet wavelet) which can provide a precise time-frequency representation of a signal [2, 3].

Layer Wise Relevance Propagation:. The LRP algorithm back-propagates through a network to decompose the final output decision, $f(\boldsymbol{x})$ [4, 5]. Briefly, a trained model's activations, weights and biases are first obtained in a forward pass through the network. Secondly, during a backwards pass through the model, LRP attributes relevance to the individual input nodes, layer by layer. For example R_k denotes the relevance for neuron k in layer $^{(l+1)}$, and $R_{j\leftarrow k}$ defines the share of R_k that is redistributed to neuron j in layer $^{(l)}$. The fundamental concept underpinning LRP

is that the conservation of relevance per layer, which can be denoted as:

$$\sum_{j} R^{(l)}_{j \leftarrow k} = R^{(l+1)}_{k} \tag{1}$$

The LRP algorithm initiates at the model output and iterates over all layers in the model in a backwards pass until the relevance scores R_i for all inputs of x_i are computed. Relevance values $R_i > 0$ signify components x_i which represent the presence of the predicted class, while conversely $R_i < 0$ contradict the prediction of that class. $R_i \approx 0$ indicate inputs x_i which have little or no influence to the model's decision.

It has been demonstrated that the combination of different rules throughout a network yields the most faithful and understandable LRP explanations [6]. As such, we applied a composite rule (LRP-CMP) with LRP-γ applied to the shallower convolutional layers, LRP-ϵ rules (ϵ=$\{1e^{-9}, 1e^{-3}, 10\}$) in the middle of the network, and LRP-0 applied to the final linear classification layer.

Visually interpreting self-supervised attribution:. To visually interpret self-supervised attribution, we compared the raw time-series accelerometery with the analogous self-supervised task transformed data. In in the panel plot depicted in figures 4 and 5, the top rows represent the 3-axis accelerometer trace for each channel: $(\boldsymbol{a}_x, \boldsymbol{a}_y, \boldsymbol{a}_z)$; the second rows depict the top view of the continuous wavelet transform (CWT) scalogram of $\|\boldsymbol{a}\|$, which is the absolute value of the CWT as a function of time and frequency. The bottom rows denote the relevance values (R_i) attributed using LRP. Red and hot colours identify input segments where $R_i > 0$ (contribution to a

class prediction), whereas blue and cold hues identify $R_i < 0$ (contradicting a class prediction), while black represents ($R_i \approx 0$) inputs which have little or no influence to the model's decision. Square patches over the raw accelerometer trace correspond to the video frames depicted above each panel plot.

Evaluating XAI algorithm faithfulness:. In order to test the faithfulness of an explanation provided by an XAI framework, a sample-masking experiment was performed, comparing some popular XAI models and LRP parameters options. Briefly, to conduct a permutation analysis, the most relevant samples identified from each XAI algorithm were cumulatively masked (imputation with random Gaussian noise), from most relevant to least relevant [4]. As such, the faster the accuracy of the model decreases with the number of masked samples, the more faithful the explanation method is with respect to the decision of the neural network.

Permutation tests were performed in random batches containing correctly identified duplicate original and augmented samples (AoT + permutation + TW) from 1000 subjects in the out-of-sample test set sampled in the *UK Biobank*. The mean degradation in the self-supervised learning prediction through our permutation experiment is shown in Supplementary Figure 3. We compared various LRP parameter options, LRP-0, LRP-ϵ, LRP-CMP, as well as some popular off-the-shelf XAI attribution frameworks, such as saliency mapping [7], Guided Backpropagation (GBP) [8] and Integrated Gradients (IG) [9].

2. Notes

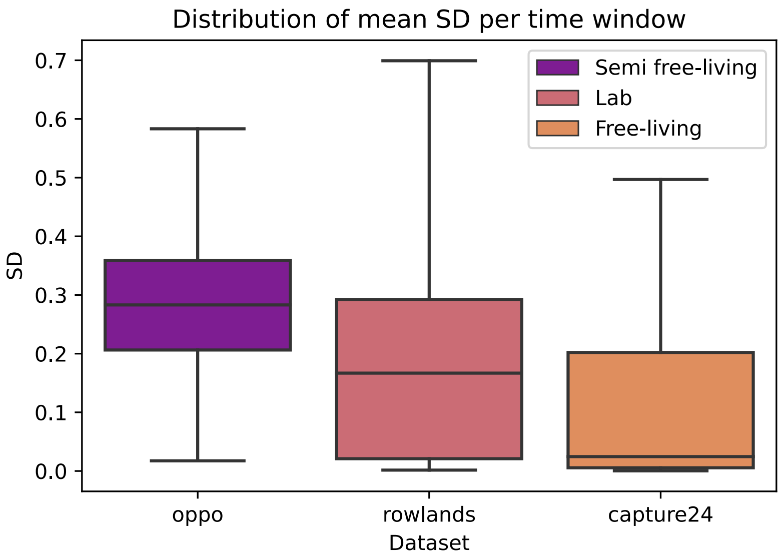

Supplementary Figure 1: Data collected from a free-living environment tends to have more stationary periods than the data collected in a lab environment. Median lines are drawn. The box boundary shows the upper and lower quarterlies.

Supplementary Table 3: Downstream human activity recognition performance (subject-wise F1 (±SD)) when using different self-supervised learning tasks after fine-tuning: Arrow of the Time (AoT), Permutation (P), Time warping (TW). The tasks were trained using the same 1,000 UK Biobank participants.

Task	Capture-24	Rowlands	WISDM	REALWORLD	Opportunity	PAMAP2	ADL
#Subjects	152	55	46	14	4	8	7
#Samples	573K	36K	28k	12k	3882	2869	635
AoT	.671 ± .094	.565 ± .120	.767 ± .124	.750 ± .084	.582 ± .054	.715 ± .036	.754 ± .157
Permutation	**.721 ± .093**	**.783 ± .099**	**.778 ± .109**	.766 ± .063	**.588 ± .076**	**.750 ± .057**	**.810 ± .097**
TW	.715 ± .093	.776 ± .110	.767 ± .120	**.772 ± .073**	.584 ± .064	.737 ± .079	.765 ± .117

Supplementary Table 4: Test accuracies on different tasks 1000 subjects.

AoT	Permutation	Time warped
0.93	.90	.97

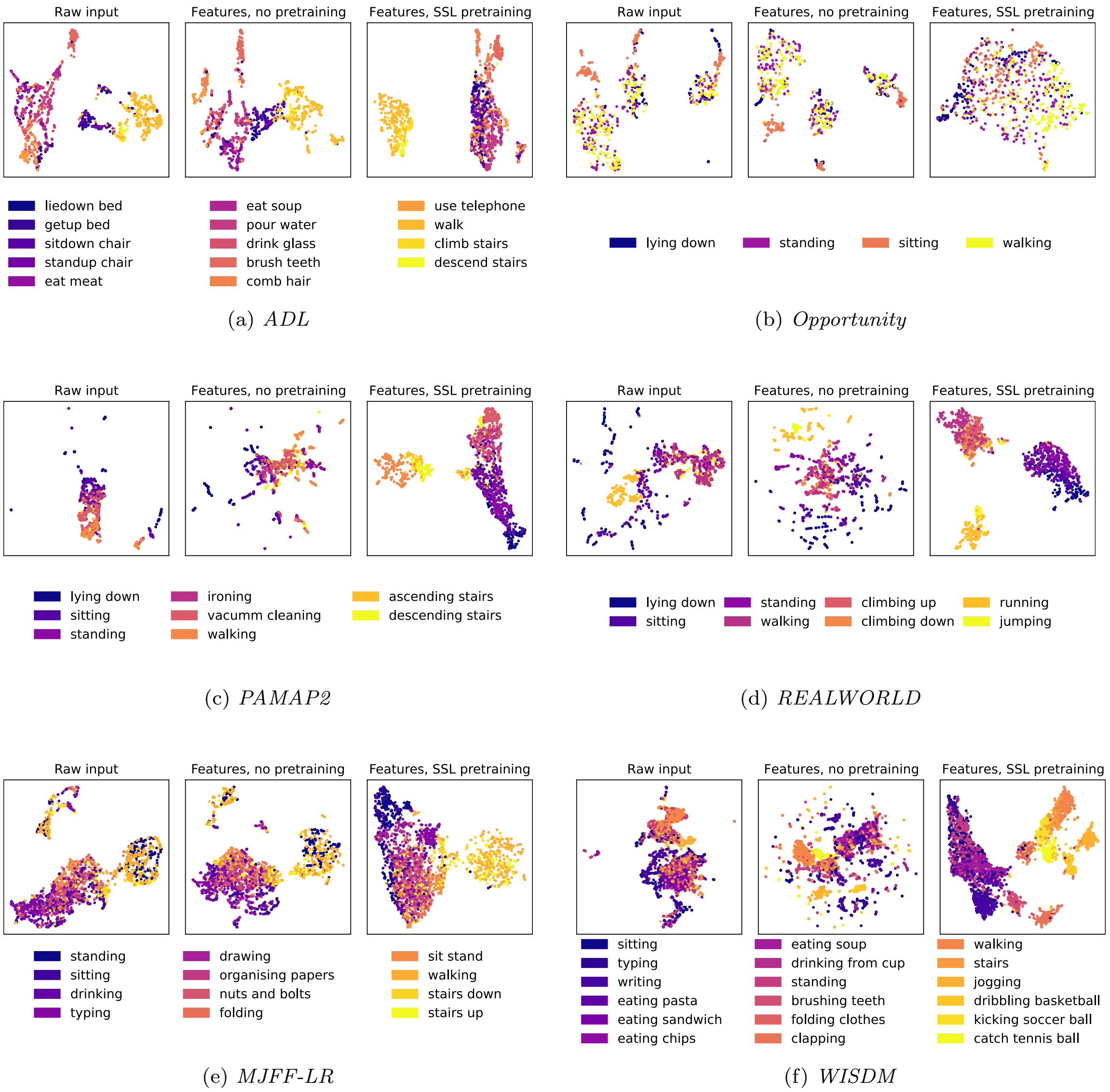

Supplementary Figure 2: Cluster analysis on raw inputs, untrained features and -pretrained features. We use color gradients to denote activity intensities. Results suggest that self-supervised-learning-derived features are better at clustering similar activities (e.g. walking, stair climbing vs. sitting, writing, typing) as well as their intensities (e.g. lying down, sitting, standing vs. jogging, sports).

2.1. Explainable Framework Performance

We observed that, for most explanation methods, removing relevant features quickly destroyed class evidence, degrading the out-of-sample test accuracy of the self-supervised model, as shown in Supplementary Figure 3. This inferred that the XAI methods had fatefully identified relevant patterns within the accelerometer signal for determining the pretext task compared to randomly mixing and masking samples in the signal. Adding random noise to the accelerometer was demonstrated to degrade model performance (which is to be expected) but at a slower rate than masking the most relevant samples first.

Furthermore, replacing the accelerometer signal with noise, cumulatively from the first time-step until the last (essentially disrupting the AoT), was found to degrade AoT-specific model accuracy at a slower rate than masking samples based on XAI relevance sorting or randomly. Note: AoT disruption was calculated from the start of the signal to the end (forward) and from the end of the signal to the start (reverse) and presented as the mean AoT disruption. As such, the the model does not appear to learn a single sequential pattern within a time-series (either forward or reverse), but instead suggests that there may be certain signal morphologies that may indicate the self-supervised learning augmentation, such as the characteristics of dynamic human motion.

2.2. Contextual LRP Examples

Supplementary Figures 5a and 5b depict further examples of contextual LRP collected during the during unscripted, repetitive, high intensity activity of playing tennis. For example, Supplementary Figure 5a depicts when the participant dropped

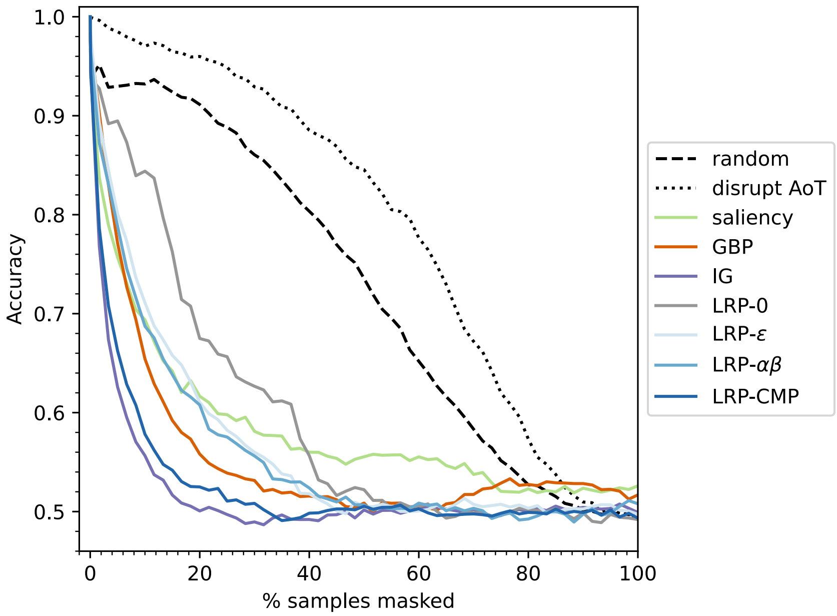

Supplementary Figure 3: Comparison of popular explainability algorithm performance on AoT + permutation + TW for 1000 random subjects in the UK Biobank out-of-sample test data, through consecutively masking samples from most relevant to least relevant. We observed that, for most explanation methods, removing identified relevant features quickly destroyed class evidence, thus degrading the out-of-sample test accuracy of the model compared to randomly masking samples.

the ball; Supplementary Figure 5b illustrates an overhead tennis serve. It was observed that LRP tended to also attribute relevance to the moments of natural human motion captured during these shots.

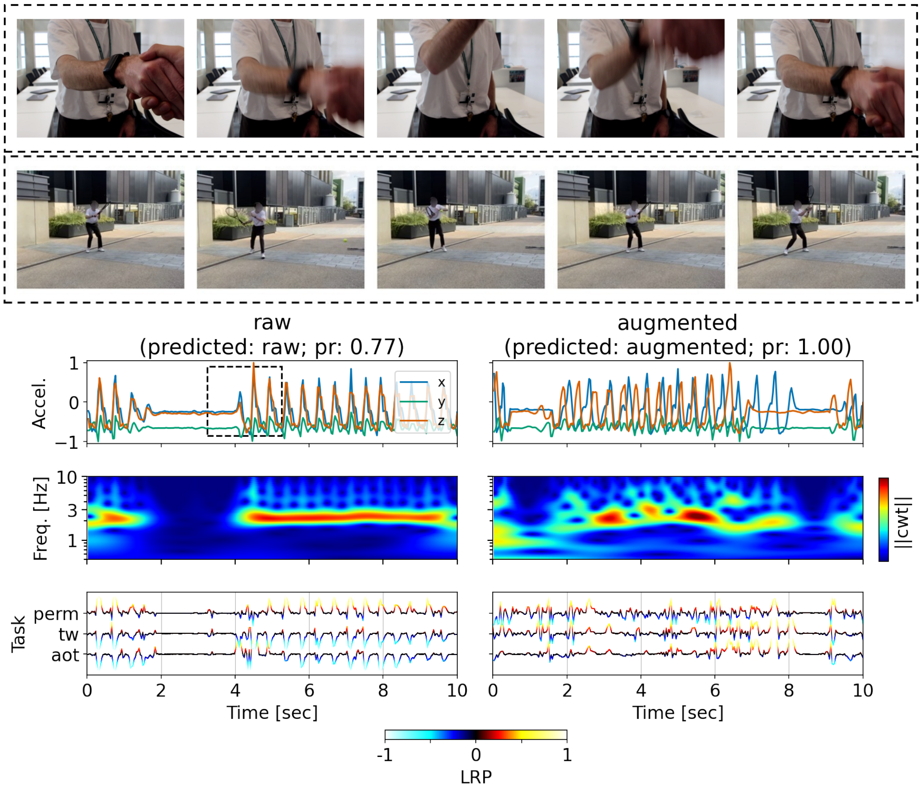

(a) Shaking hands

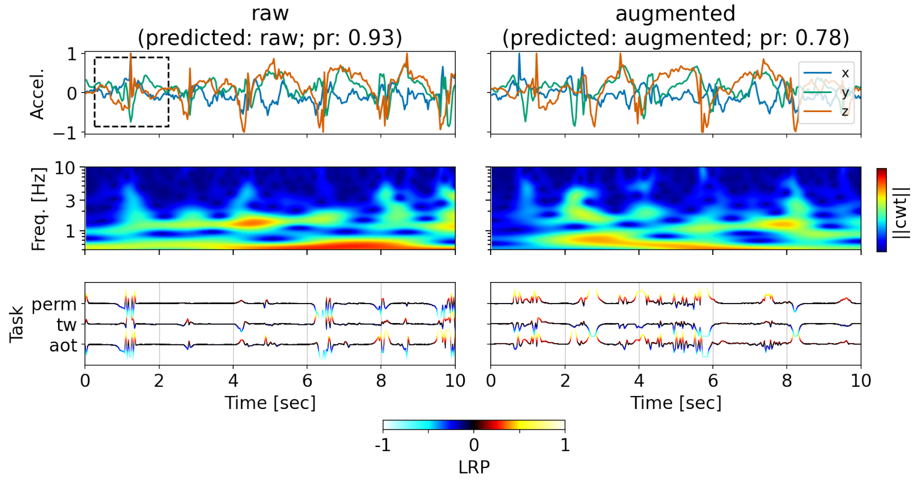

(b) Playing tennis

Supplementary Figure 4: Arrow of time + permutation + time-warped signals of (a) scripted, repetitive low intensity activities, e.g. shaking hands and (b) unscripted, repetitive high intensity activity, e.g. playing tennis. The first row shows the concurrent video frames when a participant performs an activity. The second row is the accelerometry trace. The third row is the continuous wavelet transform scalograms. The last row is the task-specific LRP attribution. Dashed patches over the acceleration trace correspond to the concurrent video video frames. Probability (Pr.) of individual transform applied to: (a) raw: AoT (0.), permutation (.68), TW (0.); augmented: AoT (1.), permutation (1.), TW (1.); (b) AoT (.03), permutation (.18) , TW (0.) ; augmented: AoT (1.), permutation (.98), TW (.36). Consent was obtained to use the subject photographs.

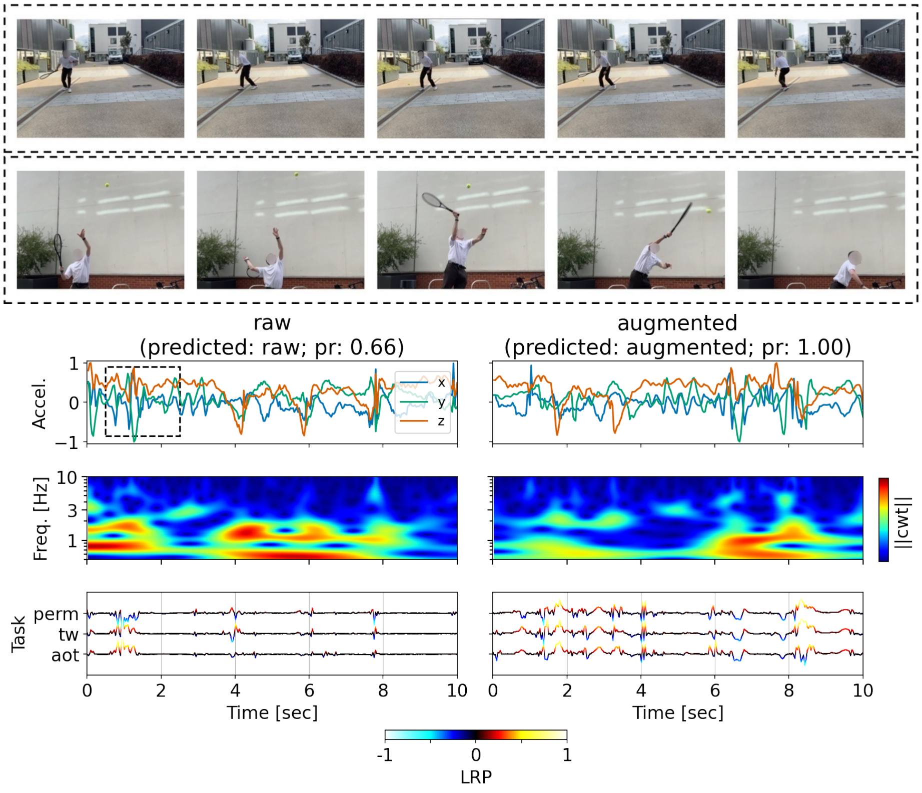

(a) playing tennis (unstructured dropping the ball)

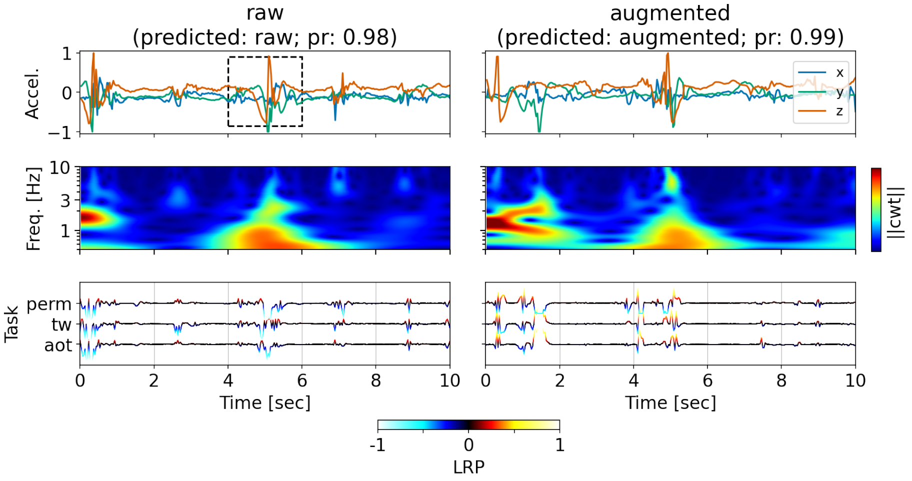

(b) playing tennis (serve)

Supplementary Figure 5: Arrow of time + permutation + time-warped signals during unscripted, repetitive high intensity intensity activity of playing tennis. The square patches over the acceleration trace correspond to the video frame depicted during when the participant (a) dropped the ball and (b) performed a tennis serve. Probability (Pr.) of individual transform applied to: (a) raw: AoT (.01), TW (0.), perm (1.); augmented: AoT (1.), TW (1.), perm (1.); (b) raw: AoT (.01), TW (0.), perm (.04); augmented: AoT (1.), TW (1.), perm (.98). Consent was obtained to use the subject photographs.

References

1. Saeed, A., Ozcelebi, T. & Lukkien, J. Multi-task self-supervised learning for human activity detection. *Proceedings of the ACM on Interactive, Mobile, Wearable and Ubiquitous Technologies* **3,** 1–30 (2019).
2. Creagh, A. P. *et al.* Smartphone- and Smartwatch-Based Remote Characterisation of Ambulation in Multiple Sclerosis during the Two-Minute Walk Test. *IEEE Journal of Biomedical and Health Informatics.* ISSN: 2168-2208 (2020).
3. Addison, P. S., Walker, J. & Guido, R. C. Time–frequency analysis of biosignals. *IEEE Engineering in Medicine and Biology Magazine* **28,** 14–29. ISSN: 0739-5175 (2009).
4. Samek, W., Montavon, G., Lapuschkin, S., Anders, C. J. & Müller, K.-R. Explaining Deep Neural Networks and Beyond: A Review of Methods and Applications. *Proceedings of the IEEE* **109,** 247–278 (2021).
5. Montavon, G., Binder, A., Lapuschkin, S., Samek, W. & Müller, K.-R. in *Explainable AI: Interpreting, Explaining and Visualizing Deep Learning* 193–209 (Springer, 2019).
6. Kohlbrenner, M. *et al. Towards Best Practice in Explaining Neural Network Decisions with LRP* in *2020 International Joint Conference on Neural Networks (IJCNN)* (2020), 1–7.
7. Simonyan, K., Vedaldi, A. & Zisserman, A. Deep inside convolutional networks: Visualising image classification models and saliency maps. *arXiv preprint arXiv:1312.6034* (2013).

8. Springenberg, J. T., Dosovitskiy, A., Brox, T. & Riedmiller, M. Striving for simplicity: The all convolutional net. *arXiv preprint arXiv:1412.6806* (2014).

9. Sundararajan, M., Taly, A. & Yan, Q. *Axiomatic attribution for deep networks* in *ICML* (2017), 3319–3328.
