# Supplementary material for: Self-supervised learning for human activity recognition using 700,000 person-days of wearable data
Source: NPJ Digit Med. 2024 Apr 12;7:91. doi: 10.1038/s41746-024-01062-3 (PMC11015005; doi:10.1038/s41746-024-01062-3)
Supplement: Supplementary file 2 — Reporting Summary [file 41746_2024_1062_MOESM2_ESM.pdf]

nature portfolio

Corresponding author(s): Aiden Doherty

Last updated by author(s): Sep 14, 2023

Reporting Summary

Nature Portfolio wishes to improve the reproducibility of the work that we publish. This form provides structure for consistency and transparency in reporting. For further information on Nature Portfolio policies, see our Editorial Policies and the Editorial Policy Checklist.

Statistics

For all statistical analyses, confirm that the following items are present in the figure legend, table legend, main text, or Methods section.

n/a	Confirmed	
☐	☒	The exact sample size (*n*) for each experimental group/condition, given as a discrete number and unit of measurement
☒	☐	A statement on whether measurements were taken from distinct samples or whether the same sample was measured repeatedly
☒	☐	The statistical test(s) used AND whether they are one- or two-sided<br>*Only common tests should be described solely by name; describe more complex techniques in the Methods section.*
☒	☐	A description of all covariates tested
☒	☐	A description of any assumptions or corrections, such as tests of normality and adjustment for multiple comparisons
☐	☒	A full description of the statistical parameters including central tendency (e.g. means) or other basic estimates (e.g. regression coefficient) AND variation (e.g. standard deviation) or associated estimates of uncertainty (e.g. confidence intervals)
☒	☐	For null hypothesis testing, the test statistic (e.g. *F*, *t*, *r*) with confidence intervals, effect sizes, degrees of freedom and *P* value noted<br>*Give P values as exact values whenever suitable.*
☒	☐	For Bayesian analysis, information on the choice of priors and Markov chain Monte Carlo settings
☒	☐	For hierarchical and complex designs, identification of the appropriate level for tests and full reporting of outcomes
☒	☐	Estimates of effect sizes (e.g. Cohen's *d*, Pearson's *r*), indicating how they were calculated

Our web collection on statistics for biologists contains articles on many of the points above.

Software and code

Policy information about availability of computer code

Data collection	No software was used
Data analysis	All the code for data processing, training the model and evaluation can be found on Github. https://github.com/OxWearables/ssl-wearables

For manuscripts utilizing custom algorithms or software that are central to the research but not yet described in published literature, software must be made available to editors and reviewers. We strongly encourage code deposition in a community repository (e.g. GitHub). See the Nature Portfolio guidelines for submitting code & software for further information.

Data

Policy information about availability of data

All manuscripts must include a data availability statement. This statement should provide the following information, where applicable:

- Accession codes, unique identifiers, or web links for publicly available datasets
- A description of any restrictions on data availability
- For clinical datasets or third party data, please ensure that the statement adheres to our policy

The UK Biobank accelerometery dataset used for training can be requested by application: https://www.ukbiobank.ac.uk/enable-your-research/register. All the other evaluation datasets can be downloaded via https://github.com/OxWearables/ssl-wearables. The MJFF-LR study can be accessed via https://www.synapse.org/#!Synapse:syn20681023/wiki/594686 after registration. The Rowlands dataset can be requested by contacting Alex Rowlands directly.

Research involving human participants, their data, or biological material

Policy information about studies with human participants or human data. See also policy information about sex, gender (identity/presentation), and sexual orientation and race, ethnicity and racism.

Reporting on sex and gender	Information regarding sex was obtained for each dataset used where possible.<br>The findings apply to both males and females.<br>No special consideration was given to sex in the study design.<br>Sex was determined from self-reporting.<br>Overall, around data samples from 60,000 females and 45,000 males were included.<br>We did not perform sex-specific analysis as sex has not been identified as a key factor that influences the performance of human activity recognition models.
Reporting on race, ethnicity, or other socially relevant groupings	NA
Population characteristics	Mostly, data from health participants were obtained. One dataset consists of patients with diagnosed Parkinson's disease. The datasets were collected in Europe and the USA.
Recruitment	This paper performs secondary data analysis and did not recruit any participant.
Ethics oversight	The data used (project ref 21/NW/0157) is covered by ethical approval from the NHS National Research Ethics.

Note that full information on the approval of the study protocol must also be provided in the manuscript.

Field-specific reporting

Please select the one below that is the best fit for your research. If you are not sure, read the appropriate sections before making your selection.

☒ Life sciences ☐ Behavioural & social sciences ☐ Ecological, evolutionary & environmental sciences

For a reference copy of the document with all sections, see nature.com/documents/nr-reporting-summary-flat.pdf

Life sciences study design

All studies must disclose on these points even when the disclosure is negative.

Sample size	The sample size has already been determined by the collected data as this paper performs secondary data analysis.
Data exclusions	We excluded data from participants that did not have perform all the activities in small datasets.
Replication	The results of this paper have been replicated by at least two authors successfully.
Randomization	NA
Blinding	NA

Reporting for specific materials, systems and methods

We require information from authors about some types of materials, experimental systems and methods used in many studies. Here, indicate whether each material, system or method listed is relevant to your study. If you are not sure if a list item applies to your research, read the appropriate section before selecting a response.

Materials & experimental systems

n/a	Involved in the study
☒	☐ Antibodies
☒	☐ Eukaryotic cell lines
☒	☐ Palaeontology and archaeology
☒	☐ Animals and other organisms
☒	☐ Clinical data
☒	☐ Dual use research of concern
☒	☐ Plants

Methods

n/a	Involved in the study
☒	☐ ChIP-seq
☒	☐ Flow cytometry
☒	☐ MRI-based neuroimaging
